# Transcriptional and epigenetic regulation of microglia in maintenance of brain homeostasis and neurodegeneration

**DOI:** 10.3389/fnmol.2022.1072046

**Published:** 2023-01-09

**Authors:** Shashank Kumar Maurya, Suchi Gupta, Rajnikant Mishra

**Affiliations:** ^1^Biochemistry and Molecular Biology Laboratory, Department of Zoology, University of Delhi, New Delhi, India; ^2^Tech Cell Innovations Private Limited, Centre for Medical Innovation and Entrepreneurship (CMIE), All India Institute of Medical Sciences, New Delhi, India; ^3^Biochemistry and Molecular Biology Laboratory, Department of Zoology, Banaras Hindu University, Varanasi, India

**Keywords:** microglia, brain homeostasis, transcription factors, epigenetic changes, brain immunity, neurological diseases

## Abstract

The emerging role of microglia in brain homeostasis, neurodegeneration, and neurodevelopmental disorders has attracted considerable interest. In addition, recent developments in microglial functions and associated pathways have shed new light on their fundamental role in the immunological surveillance of the brain. Understanding the interconnections between microglia, neurons, and non-neuronal cells have opened up additional avenues for research in this evolving field. Furthermore, the study of microglia at the transcriptional and epigenetic levels has enhanced our knowledge of these native brain immune cells. Moreover, exploring various facets of microglia biology will facilitate the early detection, treatment, and management of neurological disorders. Consequently, the present review aimed to provide comprehensive insight on microglia biology and its influence on brain development, homeostasis, management of disease, and highlights microglia as potential therapeutic targets in neurodegenerative and neurodevelopmental diseases.

## 1. Introduction

In 1932, del Rio-Hortega coined the term “microglial cell” to define a small group of mesodermally-derived, non-neuronal, phagocytic, non-astrocytic, and migratory cells in the central nervous system (CNS). Microglial cells are distinct from macroglial cells, which include oligodendroglia (del Rio-Hortega, 1932). Microglia contribute toward neurogenesis, synaptogenesis, and maintenance of brain homeostasis (Nayak et al., 2014). Microglial cells also secrete growth factors for restoring neurons and cause phagocytosis of cell debris during neurological diseases (Fu et al., 2014). In response to acute inflammation, microglial cells enhance brain inflammation by releasing nitric oxide (NO), reactive oxygen species (ROS), and pro-inflammatory cytokines such as TNF-α, and IFN-γ, which promote recruitment of lymphocytes to damage sites from the blood. These lymphocytes take over the function of microglia and maintain brain homeostasis (London et al., 2013; Skaper et al., 2018). While regulating brain homeostasis during healthy and disease conditions, microglia undergo alteration at transcripts and protein levels and show different morphological changes. However, uniform consensus on characterization of microglia morphologies is still under debate (Paolicelli et al., 2022). Since microglia play an active role in the pathophysiology of a number of CNS diseases. Therefore understanding microglial mechanisms of action in regulation of brain physiology is essential for the development of therapeutic modality.

During normal aging, neuroinflammation, and age-related disease, microglia are hypo-motile, chronically express pro-inflammatory signaling molecules and burdened with lysosomal cargo (Olmedillas Del Moral et al., 2019). Microglia bind to the soluble and membrane-bound mediators, regulate the local microenvironment of neurons and astrocytes, neurogenesis, synaptic pruning, support neurite formation, the outgrowth of dopaminergic axons, and laminar structure of the cortex (Andoh and Koyama, 2021; Maurya et al., 2021). Microglia also mediate synaptic transmission, synaptic structures modification or elimination (Akiyoshi et al., 2018). They interact with neurons *via* various signaling molecules including transforming growth factor-β (TGF-β), brain-derived neurotrophic factor (BDNF), and complement proteins including CR3 and C1q (Cornell et al., 2022). Epigenome and genome-wide transcriptome studies have shown that microglia are different from other glial cells and tissue macrophages (Cuadros et al., 2022). A number of microglia specific signature genes have been identified to be involved in diverse functions of microglia. Similarly, multiple key transcription factors such as Spi1, Sall1, Mef2c, and Mafb regulate microglial transcriptomes. Epigenetic modulations in the enhancer repertoire of target genes also shape immune memory in microglia (Butovsky and Weiner, 2018; Yeh and Ikezu, 2019). However, molecular mechanisms and the consequences of restoring microglial functions in neurodegenerative diseases are poorly understood. Further, understanding the various facets of microglia biology will facilitate the early detection, and management of neurological diseases. Therefore, the present review intends to present transcriptional and epigenetic factors mediated regulation of microglia functions in maintenance of brain homeostasis in normal and disease conditions.

## 2. Origin of microglia

In 1932, del Río Hortega, nervous tissue preparation using silver-carbonate staining showed a small cell body structure with various ramified cell processes. These cells were further described as microglial cells, differentiating them from the astrocytes and oligodendrocytes (macroglia). Till 1990s, scientists discussed heavily regarding ectodermal or mesodermal origin of microglia (Ginhoux et al., 2013). Further, fate mapping experiments showed that microglia have monocytic blood origin (Ginhoux et al., 2010). Mice deficient in Pu.1, a key regulator of hematopoietic development were reported devoid of microglia (Mckercher et al., 1996; Iwasaki et al., 2005; Beers et al., 2006). The Pu.1 and stem cell leukemia/T-cell acute lymphoblastic leukemia 1 (Scl/Tal-1) showed to regulate the development of erythromyeloid progenitors in the yolk sac of the mouse embryo at 8.5 post conception (E8.5; Mcgrath et al., 2015; Perdiguero and Geissmann, 2016). The subset erythromyeloid progenitor’s cells matures into CX3CR1^+^ cells and become microglial progenitors in the yolk sac and migrates into the brain between E9.5 and E14.5 (Rigato et al., 2011). Similarly, at around 4.5 to 5.5 gestational weeks, microglia invade the forebrain (Monier et al., 2007; Verney et al., 2010; Menassa and Gomez-Nicola, 2018) and the major entry and circulation starts around 16 weeks (Rezaie and Male, 1999; Rezaie et al., 2005; Verney et al., 2010; Menassa and Gomez-Nicola, 2018). After postnatal development, the microglia are observed to reside in the brain and regulate its population size in normal healthy brain by self-renewal capacity of CNS (Ginhoux et al., 2010; Ginhoux and Guilliams, 2018). Thus, microglia are brain specific immune cells which interact with other brain cells including neurons and regulate various physiological activity in the brain during normal and disease conditions.

## 3. Microglia and its interaction with brain cells

### 3.1. Neurons

Although microglia have been known for a long time to play an important role in fostering inflammatory responses in the CNS, it is now evident that these functions, particularly in the healthy brain, are much more diverse. The functional repertoire of microglia also includes biochemical homeostasis maintenance, maturation of developing neuronal circuits, and their reorganization. There is mounting evidence that neurons inform microglia about their status and are therefore involved in regulating microglial activation and motility, whereas microglia also regulate neuronal activities (Umpierre and Wu, 2021). It has been established that microglia can detect neuronal activity, modulate neuronal function, and recognize neuronal damage at an early stage (Ronzano et al., 2021). In addition, numerous studies have shown that microglia interact with neurons at the neuronal soma, a function that is dependent on purinergic signaling and may aid in neuroprotection (Illes et al., 2020; Ronzano et al., 2021). Initial segment of the axon is another site of interaction between microglia and neurons. Here, microglial processes overlap this segment and their responses to neuroinflammation may vary (Baalman et al., 2015; Benusa and Lafrenaye, 2020; Ronzano et al., 2021). The bi-directional communication by various ligands and receptors are essential for cross-talk between neurons and microglia in maintaining brain homeostasis which gets altered in disease conditions.

#### 3.1.1. The signaling mechanism for microglia–neuron interactions

**Figure 1:**
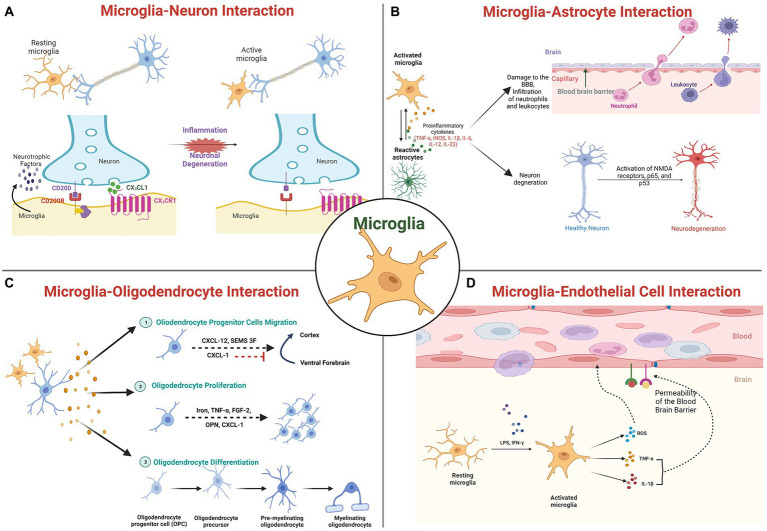
Molecular interaction of microglia with brain cells: **(A)** Microglia-Neuronal interactions; **(B)** Microglia-Astrocyte interaction; **(C)** Microglia-Oligodendrocyte interactions; and **(D)** Microglia-Cerebrovascular endothelial cells interactions.

Microglia express a wide variety of neurotransmitter receptors, including glutamatergic receptors, serotonin receptors, etc., whose stimulation affects essential functions, such as cytokine production, cellular motility, and phagocytosis. The molecules released by microglia activate respective neuronal receptors, enabling microglial control of neurotransmission (Figure 1A). In response to pathological stimuli, neuronal regulatory mechanisms are compromised. These alterations disrupt the specific communication pathways between microglia and neurons, thereby disrupting the neuronal circuits associated with functions. Several molecules and receptors with specific roles in microglia-neuronal interactions have been identified.

Purinergic signaling evolves as a central system in the kinetics of neuron-to-microglia interaction and influences microglial behavior *via* interactions with other systems (Inoue, 2017; Szepesi et al., 2018; Calovi et al., 2019). In pathology, it is known that this signaling is essential for epileptogenesis. Microglia in necrotic regions have an amoeboid shape and respond more rapidly to purinergic stimuli as indicated by experimental seizures and temporal lobe epilepsy patients, indicating an elevated expression of purinergic receptors (Morin-Brureau et al., 2018).

CX3CL1, also known as Fractalkine, is a transmembrane chemokine that is secreted by neurons in the central nervous system (CNS) and signals through its unique receptor, CX3CR1, which is expressed by microglia (Chen et al., 2016). It has been demonstrated that the CX3CL1/CX3CR1 signaling pathway regulates bidirectional interaction between neurons and microglia and maintains neural–immune communication (Paolicelli et al., 2014; Mecca et al., 2018). In particular, mice with Cx3cr1 deletion have been reported to display abnormal pruning of dendritic spine, synapse maturation, altered functional connectivity, and behavioral defects (Paolicelli et al., 2011; Zhan et al., 2014). The loss of CX3CR1 signaling has been linked to neuronal degeneration, as observed in PD and ALS animal models (Pawelec et al., 2020). In ischemic mice brain, silencing of Cx3cr1 using siRNA showed to reduces microglia activation, white matter lesions, and cognitive deficits and attenuate the elevated expression levels of Cx3cr1, p38Mapk, Pkc, Tnf-α, Il-1, and Il-6 (Liu et al., 2015). Further the same group has also reported that exogenous Cxc3l1 administration to BV2 microglial cells treated with oxygen–glucose deprivation (OGD) showed increased expression of cytokines IL-1 and TNF-α, which got attenuated by silencing Cxc3r1 or by inhibiting p38Mapk indicating putative role of CX3CRL1/CX3CR1 axis in pathophysiology of brain ischemia (Liu et al., 2015). Clinical studies demonstrate an association between CX3CR1 polymorphisms and an early progression of ALS symptoms and delayed AD, as well as a shorter survival time. Additionally, CX3CR1 variants have been linked to an increased risk of neurodevelopmental disorders, such as ASD and schizophrenia (Lopez-Lopez et al., 2014; Hanger et al., 2020; Puntambekar et al., 2022).

In addition, complement receptors regulate a variety of microglial functions, including motility, phagocytosis, and cytokine release. These processes are relevant to the refinement of brain circuitry. Neurodegenerative disorders, which are characterized by a massive loss of neurons, an abundance of misfolded proteins, and adverse synaptic changes, alter these functions (Hickman et al., 2018). Intriguingly, both human and rodent neurodegenerative disease models exhibited a massive activation and overexpression of complement factors and receptors. Microglia are reported to express high levels of the complement system receptors C1q, C3, and C5 (CR3 and CR5) under pathological conditions or early in postnatal development (Hong et al., 2016; Petralla et al., 2021). TREM2, an innate immune receptor that is also expressed on the membrane of microglia and is involved in numerous processes, including activation, proliferation, clustering, and survival, is another signaling mechanism. Upon binding with ligands such as LPS, DNA, phospholipids, Trem2 receptor gets activated and inhibit inflammatory response mediated by TLR and promote apoptotic body clearance by enhancing microglial migratory and phagocytic activity (Zheng et al., 2018). Human genetic studies that link mutations in the TREM2 gene to an increased risk of neurodegenerative disorders provide support for the significance of TREM2 in neurodegeneration (Yaghmoor et al., 2014). Likewise, neurodevelopmental disorders exhibit altered TREM2 pathway activity. Autistic patients have been found to have reduced levels of TREM2 (Filipello et al., 2018). Furthermore, the interaction between the neuronal CD200 glycoprotein and the microglial CD200R receptor has also been reported to facilitate neuron–microglia communication, resulting in the formation of the CD200-CD200R complex for the regulation of brain inflammatory pathology (Lyons et al., 2007; Walker and Lue, 2013). Intriguingly, a decrease in expression of microglial CD200R and neuronal CD200 was observed associated with the aging process, indicating abnormal microglial activation may be a cause of susceptibility to neuroinflammation and neurodegenerative diseases (Walker and Lue, 2013). Despite being less conventional, neurotrophins such as BDNF, NGF, etc. are also involved in crosstalk between microglia and neurons, as microglia also secretes such factors and both neurons and microglia express their receptors (Szepesi et al., 2018). Accumulating evidence indicates that the microglial endocannabinoid system functions as a “non-canonical” signaling mechanism in the management of neuron–microglia crosstalk, thereby assisting microglia in regulating brain inflammation (Castillo et al., 2012; Scipioni et al., 2022). CB1Rs are predominantly expressed in neurons, whereas CB2Rs are characteristic of microglia, especially during neuroinflammation (Komorowska-Müller and Schmöle, 2021). In healthy brains, eCB production by microglia was reported consistently low and elevated in diseased brains. eCB signaling in microglia is distinguished by particular degradation pathways (Tanaka et al., 2020). Specifically, inhibiting them reduces neuroinflammation without affecting eCB signaling in neurons.

#### 3.1.2. Exosomes in microglia–neuron interaction

In addition to these signaling mechanisms for microglia-neuronal interaction, accumulating evidence suggests that nano-vesicles known as exosomes with sizes ranging from 40 to 100 nm, secreted by microglia, may serve as important intercellular signaling mediators (Guo et al., 2021). These exosomes exert their effects by transporting specific cargos, such as proteins, microRNAs (miRNAs), and messenger RNAs (mRNAs) for cell to cell communication. It has been demonstrated that microglial exosomes can be transported to and taken up by neurons, which may be beneficial or detrimental to CNS diseases (Liu et al., 2019; Guo et al., 2021). miRNAs, mRNAs, proteins, etc. can be incorporated into multivesicular bodies (MVBs) intracellularly and then secreted by microglial exosomes. MVBs are formed by the inward protrusion of the endocytic membrane, later fuse with plasma membrane to release exosomes. Microglial cells, on the other hand, release microvesicles (MV), which are larger than 200 nm in diameter, through plasma membrane budding (Brites and Fernandes, 2015; Scott, 2017). Exosomes are predominantly enriched with receptors and kinases, indicating their role in cellular signaling. The presence of mitochondrial, centrosomal, and ribosomal proteins on MVs suggests their roles in protein translation. In addition, microglial exosomes are involved in antigen presentation, such as the transfer of antigens, indicating a functional role in immune response and are essential for regulating the brain-immune system interaction (Kalluri and LeBleu, 2020; Guo et al., 2021; Hazrati et al., 2022). Studies have revealed that neurodegenerative disease-related proteins are transported by microglial exosomes. The ability of activated microglia to release exosomes containing misfolded proteins such as α-synuclein, tau, Aβ, and cytokines has been documented (Španić et al., 2019; Aires et al., 2021). Additionally, they contain the insulin-degrading enzyme (IDE), which can degrade the Aβ peptide. Importantly, upon activation, microglial cells exhibit a high degree of plasticity, thereby releasing exosomes affecting the distant brain regions. The activated microglia are suggested to spread misfolded proteins either through membrane leakage or exosomes after migrated cells die, making them an efficient disease transporter (Aires et al., 2021; Guo et al., 2021). The relationship between microglial exosomes and the pathogenesis and progression of neurodegenerative diseases has garnered considerable attention. However, exosome related molecular mechanisms of microglia functions in pathogenesis and therapeutics of neurodegenerative diseases are still under investigation.

### 3.2. Non-neuronal cells

As mentioned previously, microglia cells also exert their primary functions toward non-neuronal cells such as astrocytes, oligodendrocytes, vascular cells, etc., and a great deal of research focuses on their dyadic interactions. Recently, crosstalk between microglia and astrocytes has dominated glial research. Emerging evidence indicates that signals derived from microglia and astrocytes are the functional determinants (Figure 1B). Microglia and astrocytes develop bidirectional communication and autocrine feedback for tight reciprocal alteration during CNS insult or injury by releasing diverse signaling molecules (Matejuk and Ransohoff, 2020). Interactions between microglia and astrocytes are fundamental to neuronal functions and dysfunctions (Ricci et al., 2009). Microglia can modulate neuronal activity and receive information from astrocytes since these astrocytes express membrane receptors for all known neurotransmitters (Pascual et al., 2012). Astrocytes reduce microglial activation by upregulating TGF-β and Galectin-1, thereby decreasing antigens presentation-related factors secretion such as pro-inflammatory cytokines, NO, ROS, and TNF-α (Xu et al., 2020). The significance of Galectin-1 secretion has been hypothesized based on the improved clinical outcomes in a mouse model of experimental autoimmune encephalomyelitis (EAE; de Jong et al., 2020). IL-1, which is predominantly produced by microglia, is closely linked to neuronal disorders, increases astrocyte neurotoxicity, and may also contribute to their protective function (Hansen et al., 2018). Microglia express abundant purinergic receptors, and ATP released by astrocytes in response to a local injury activates local microglia (Franke et al., 2012). Astrocyte Ca + ion waves, which are involved in synaptic activity and regulate microcirculation, glutamate production, and release, and ion homeostasis, also spread to microglia (Hung et al., 2010).

Oligodendrocytes are non-neuronal cells that produce myelin for the myelination process, a crucial step in the development of the CNS. Myelin sheath is composed of oligodendrocyte plasma membranes with specific protein composition and multiple layers. Each oligodendrocyte produces multiple myelin internodes that insulate numerous axons in their vicinity, thereby facilitating faster action potential conduction. The evidence of crosstalk between microglia and oligodendrocytes in the repair, genesis, and regulation of myelin-producing cells has also been reported (Peferoen et al., 2014; Figure 1C). Oligodendrocytes have the ability to regulate microglial activity through the production of chemokines, cytokines, and chaperokines. By inhibiting apoptosis of oligodendrocyte precursors and promoting their differentiation, non-activated microglia promote oligodendrocyte survival and increases the number of mature oligodendrocytes (Kalafatakis and Karagogeos, 2021). Activation of the p38 MAPK in microglia mediates growth factor production and is essential for apoptosis of oligodendrocytes in SCI (Yune et al., 2007). Further, the report also suggests microglia activation inhibits oligodendrocyte progenitor cell (OPC) proliferation and induces apoptosis (Yune et al., 2007; Peferoen et al., 2014). Similar observations have been made in MS lesions, indicating a correlation between oligodendrocyte damage and microglial activity in MS. After spinal cord injury, microglia have been observed in close proximity to dying oligodendrocytes and they may promote oligodendrocyte survival (Miller et al., 2007). This proximity after injury suggests a mechanism by which microglia may influence the survival of oligodendrocytes and OPCs, highlighting the dual role that microglia may play in CNS injury.

Furthermore, Cerebrovascular endothelial cells (CVEs) are well known to create physical and functional barriers, separating CNS from peripheral circulation by forming brain microvasculature, and are in permanent bidirectional communication with microglia cells (Figure 1D). Microglia are attracted by a breach in the BBB, and its activation cause increased production of pro-inflammatory mediators such as TNF-α and IL-1b, as well as inducible nitric oxide synthase (iNOS) expression level (Wang et al., 2015; Kang et al., 2020) leading to neuroinflammation and progression of neurodegenerative diseases (Takata et al., 2021). Mice lacking CD200R produce more Tnf-α in response to the gram-negative bacterial endotoxin lipopolysaccharide (LPS; Walker and Lue, 2013). CVEs contain TNF receptors (TNFRs), to which TNF-α binds. After ischemia and in humans with neurodegenerative diseases, brain TNF-α levels rise, and this rise is largely due to activated microglia. Microglia are found in close proximity to vascular endothelial cells (VECs), indicating a possible role in promoting the formation of healthy CNS vascular networks and regulating the process in the adult brain (Zhao et al., 2018). Two angiogenic factors, ephrin-A3 and ephrin-A4, were highly expressed in CVEs treated with microglial culture supernatant, indicating that microglia induce *in vitro* angiogenesis in brain endothelial cells (Li et al., 2014). Similarly, Stat3, which is predominantly expressed by activated microglia and other macrophages 24 to 72 h after cerebral ischemia, is said to promote angiogenesis by regulating the migration and proliferation of CVEs. Inhibiting Stat3 phosphorylation within 72 h of cerebral ischemia decreased lesion size (Hoffmann et al., 2015). CVEs also express MMP-3 (matrix metalloproteinases-3), which plays a crucial role in disruption of blood–spinal cord barrier (BSCB) following spinal cord injury (SCI), contributing to activation of the microglia and enhanced inflammation (Behl et al., 2021).

## 4. Microglia and their association with learning, memory, and cognition

Synapse remodeling is an ongoing process in the adult brain that alters synaptic connections and is required for encoding memory in the neural circuit (Südhof, 2018). The mechanisms that contribute to remodeling synapses are essential for flexible learning and memory (Benson and Huntley, 2012). Microglia surveillance has been linked to synaptic maturation. Microglia has been reported to actively engulf synaptic material and play an important role in synaptic pruning and hence microglia surveillance has been associated with synaptic maturation. Further, it has been proposed that deficits in microglia function may lead to synaptic abnormalities in some neurodevelopmental disorders (Paolicelli et al., 2011). It has been reported that microglia make activity-dependent contact with synapses in order to regulate synaptic density and connectivity (Andoh and Koyama, 2021). Multiple studies have demonstrated that microglia along with neural ensemble connectivity and memory strength, also regulate memory quality (i.e., the ability to distinguish between similar contexts; Nguyen et al., 2020; Wang et al., 2020). The removal of microglia leads to changes in organization and activity of glutamatergic synapses (Basilico et al., 2022). Microglial autophagy is also shown to regulate synapses and neurobehavior. Deletion of atg7, which is vital for autophagy, from myeloid cell-specific lysozyme M-Cre mice resulted in increased dendritic spines, synaptic markers, altered connectivity between brain regions, social behavioral defects and repetitive behaviors. Further, atg-7 deficient microglia showed impaired synaptosome degradation whereas increase in immature dendritic filopodia in neurons were observed in neurons co-cultured with atg-7 deficient microglia (Kim et al., 2017). Microglial Bdnf is shown to increase phosphorylation of a key mediator of synaptic plasticity in neurons, i.e., tropomyosin-related kinase receptor B and regulate learning and memory *via* BDNF signaling as removal of Bdnf from microglia restates deficits in multiple learning task due to microglia depletion (Parkhurst et al., 2013). CD47-SIRPα interaction and signaling has also been observed to regulate microglia mediated synaptic pruning and cognitive ability in neurodegeneration (Lehrman et al., 2018; Ding et al., 2021). During the initial pathological stage of perioperative neurocognitive disorders (PND), microglia mediated astrocyte activation leads to long-term synaptic inhibition and cognitive deficiencies (Li et al., 2020). The majority of studies have identified that IL-33 signaling *via* microglia is crucial for memory quality. Interleukin-33 (IL-33) is expressed in an experience-dependent manner by adult hippocampal neurons and defines a subset of neurons primed for synaptic plasticity. The loss of microglial IL-33 receptor or neuronal IL-33 results in altered spine plasticity, decreased newborn neuronal integration, and reduced accuracy of distant fear memories. The precision of memory and neuronal IL-33 reported diminished in aged mice, and gain of IL-33 function alleviates age-related declines in spine plasticity (Nguyen et al., 2020). Studies have shown that neuronal IL-33 directs microglia mediated engulfment of the extracellular matrix (ECM) and that its absence results in weakened ECM engulfment and ECM proteins accumulation in contact with synapses (Vainchtein et al., 2018). These findings identify a cellular mechanism by which microglia regulate experience-dependent synaptic remodeling and facilitate memory consolidation. Another study by Wang et al. (2020) reported that the C1q-dependent complement pathway is majorly involved in microglia mediated synapse elimination, and that CD55, a complement pathways inhibitor, disrupt C1q-dependent complement pathway and microglia in memory-storing engram cells in dentate gyrus (Wang et al., 2020). Another interaction is *via* the CD200/CD200R pathway, in which CD200R is expressed by microglia and CD200 is expressed by neurons (Sun et al., 2020). In the presence of amyloid beta, CD200 knockout mice exhibit greater phagocytosis than wild-type mice (Lyons et al., 2007). Apart from playing an important role in pathophysiology of neurodegeneration, the CX3CL1/CX3CR1 axis is also reported to influence synaptic plasticity and cognition. The CX3CL1 has been shown to inhibit hippocampal LTP through adenosine receptor 3 activity (Maggi et al., 2009). CX3CR1 deficiency is observed to show reduced neurogenesis, impaired synaptic plasticity and cognitive functions (Bachstetter et al., 2011; Rogers et al., 2011). The decrease in CX3CL1-CX3CR1 signaling and cognitive deficits has also been noted in Streptozotocin (STZ) induced mice (Kawamura et al., 2020). Thus, microglia protect strong, active synapses essential for learning and memory *via* this pathway. However, understanding the molecular mechanism of microglia function in regulation of learning and memory, neuronal-microglial communications in healthy and different brain pathologies still require further investigation.

## 5. Key genes and proteins involved in microglial function(s) in brain

During age-related disease, normal aging and neuroinflammation, microglia are observed to be hypo-motile, loaded with lysosomal cargo, and persistently express pro-inflammatory signaling molecules (Spittau, 2017; Pluvinage et al., 2019). Epigenome and genome-wide transcriptome studies have revealed that microglia are different from other glial cells and tissue macrophages. Further, it was identified that CX3C chemokine receptor 1 (Cx3cr1), Triggering receptor expressed on myeloid cells 2 (Trem2), Spalt like transcription factor 1 (Sall1), Transmembrane protein 119 (Tmem119), Sialic-acid-binding immunoglobulin-like lectin-h (Siglec-H), Olfactomedin-like 3 (Olfml3) and P2 purinergic receptor (P2ry12) specifically expressed in microglia (Chiu et al., 2013; Galatro et al., 2017) are involved in the regulation of its physiology and could act as a possible drug target in neurodegenerative diseases. In activated microglia, ionized binding protein1 (Iba1) contributes in actin-bundling and regulates membrane ruffling, cell migration, and phagocytosis (Ohsawa et al., 2004). The Iba1 has been reported as a more suitable marker than plasma membrane and trans-membrane-specific markers for structural analysis of microglia (Korzhevskii and Kirik, 2016). The Cx3cr1 plays an important role in microglia-neurons communication (Bolós et al., 2018). Trem2 has proven essential for maintaining microglial metabolic fitness during stress and sustaining the microglial response in pathological conditions (Ulland et al., 2017). Spalt like transcription factor 1 (Sall1), regulates microglia identity and functions (Buttgereit et al., 2016) and P2ry12 serves as chemotactic receptors, guiding the microglial cell processes movement near to local sites of CNS injury (Lou et al., 2016). The Tmem119, a protein of unknown function, serves as a marker of microglia (Bennett et al., 2016). However, Siglec-H allows histological identification of microglia and microglia-specific gene manipulation in the nervous system (Konishi et al., 2017). The Olfml3, a secreted glycoprotein proves critical in the development and functional organization of the CNS, hematopoietic system, and a new target gene of Tgf-β1/Smad2 whose expression is restricted to the microglia in the brain (Neidert et al., 2018).

Additional 30 genes (*ADAM28, ACY3, ALOX5AP, ADORA3, C1QB, CD33, C3, CIITA, CD84, CSF2RA, CPED1, FCER1G, DHRS9, FYB, HPGDS, GPR34, LAPTM5, IGSF6, P2RY13, LY86, SASH3, RASAL3, SPN, SELPLG, SUSD3, SUCNR1, TBXAS1, TLR7, SYK,* and *TREM2*) have been reported to express strongly in various healthy human brain regions (Bonham et al., 2019). However, they are vulnerable in Alzheimer’s disease (AD) *via* cell-type expression profiling tool (CellMapper; Bonham et al., 2019) and are also related to gene networks of both aging and neurodegenerative diseases (Mukherjee et al., 2019). In pre-clinical stages of AD, an increase in pro-inflammatory DAM protein expression was associated with neurofibrillary tangle and tau pathology (Rangaraju et al., 2018). Various reports have suggested the involvement of the complement production and signaling (C1, C1q, C3, CR1, Factor B, Factor D, and Properdin), NLRP3 inflammasome activation (VRAC, NLRP3 P2X7,), and TREM2/DAP12 signaling (TREM2, PLCγ2, SHIP-1, and Apolipoprotein E) in the regulation of microglial survival and proliferation, actin cytoskeleton polarization, cytoskeleton organization, stimulating microglial phagocytosis, cytokine production, immune responses, and could be potential therapeutic targets in modulating microglial functions for AD (Konishi and Kiyama, 2018; Mecca et al., 2018). The molecular mechanisms and the significance of restoring microglial functions to age-related brain dysfunction are still elusive. Further, an understanding of disease-associated microglia (DAM) heterogeneity, its key regulators, and emerging “microgliome” are also required to differentiate transcriptionally distinct and neurodegeneration-specific microglial profiles for drug discovery and clinical research (Sousa et al., 2017). Thus, the discovery and identification of specific microglia signature genes and similarly preserved networks in animal models will provide mechanistic insights into microglia function(s) in neurodegenerative diseases.

## 6. Transcriptional regulation of microglial genes in normal and disease conditions

### 6.1. Regulation of microglial genes in healthy brain

PU.1 is the most abundant ETS-domain transcription factor encoded by the *Spi1* (murine) or *SPI1* (human) gene. It activates gene expression during immune cell development by binding to a purine-rich sequence (PU box). The PU.1 is reported to continuously express from erythromyeloid progenitors (EMPs) to adulthood microglia (Butovsky and Weiner, 2018). The transcription factors PU.1 and IRF8 are known to largely regulate microglia development and functional maintenance. PU.1 deficient mice lack parenchymal microglia whereas induction of PU.1 expression in human cortical organoids leads to generation of microglia like cells (Beers et al., 2006; Cakir et al., 2022). Silencing of PU.1 shown to suppress genes response for antigen presentation, pro-inflammatory response, reduces microglia number and their ramification and phagocytosis activity (Smith et al., 2013; Huang et al., 2017; Rustenhoven et al., 2018). Chromatin Immunoprecipitation followed by sequencing reveals PU.1 binds to a number of microglia sensome genes and proposed that aberrant regulation of PU.1 target genes may lead to neurodegenerative diseases by changing microglial transcriptional network (Satoh et al., 2014).

Various studies have reported the importance of PU.1 in regulating microglia function and homeostasis (Nayak et al., 2014). However, additional research is needed to understand how various transcription factors are associated with maintaining the microglial identity and functions in the homeostatic brain.

### 6.2. Regulation of microglial genes in brain pathology

In the transgenic mouse models of AD, increased expression of markers associated with microglial activation such as CLEC7A, and Galectin-3, and decreased expression of homeostatic markers including P2RY12 suggest a switch in a specific population of microglia associated with an amyloid plaque from homeostatic to reactive state (Butovsky and Weiner, 2018; García-Revilla et al., 2022). Further, dystrophic neurites in normal aging and neurodegenerative diseases lead to a phenotype switch from homeostatic to neurodegenerative microglia (MGnD) by stimulating the TREM2-APOE pathway (Pimenova et al., 2017). APOE and TGFβ are reported to be the major upstream regulators of MGnD microglia. APOE upregulation induces gene expression of transcription factors *Bhlhe40, Tfec, Atf*, and inflammatory miR-155, leading to the inflammatory response and decreasing microglial homeostatic transcriptional factors including *Spi1, Mef2a, Mafb, Smad3* (Krasemann et al., 2017). In the SOD1 mouse model of ALS, it has been observed that targeting miR-155 restores abnormal microglia and attenuates disease (Butovsky et al., 2015). In a PS19 mice model of neurodegenerative tauopathy and AD, microglia-specific transcription factors *Irf8, Spi1,* and *Runx1* are observed to be significantly upregulated, whereas complement C3aR deletion, the complement factor C3 complement receptor that mediates neuroimmune crosstalk, decreases *Irf8, Spi1,* and *Runx1* expression and rescues tau pathology and attenuates neuroinflammation, synaptic deficits, and the deleterious effect (Lian et al., 2015; Hong et al., 2016; Litvinchuk et al., 2018).

Aging showed to have an influence on microglia. The expression of pro-inflammatory genes in adult microglia increases with maturation and aging indicating reduced plasticity and a reactive state in the adult stage. In aging mice, transcripts of the microglial sensome required for sensing endogenous ligands, such as *Gpr34, Fcrl1, P2ry12, Trem2,* and *Dap12*, were downregulated, whereas genes involved in microbe recognition and host defense system, such as *Cd74, Tlr2, Ltf, Cxcl16,* and *Clec7a* were upregulated (Boche and Gordon, 2022). The age-dependent transcriptional module including *SPI, IRF8, RUXN1,* and *TAL1* is reported to regulate microglial markers (Wehrspaun et al., 2015). Additional analysis suggested a significant correlation between TLRs for communication with pathogen-associated molecular patterns, microglia surface receptors (P2RY12, CX3CR1, and TREM2), and aging, highlighting the age-dependent change in microglial plasticity (Kierdorf and Prinz, 2013; Wehrspaun et al., 2015). Recent research by Olah et al. suggests that the APOE2 haplotype protects human microglia from the increased expression of an aging gene set. In aged microglia, their findings revealed upregulation of *CD33, INPP5D, MS4A4A, SORL1,* and *TREM2*, but no change in PU.1. The discrepancy may be explained by the fact that the average age of the postmortem tissue donors was 95 years older than in previous studies and that their cohort consisted primarily of females (Olah et al., 2018).

NF-kB activation in microglia has also been shown to be associated with the development of a classic M1 pro-inflammatory phenotype (Orihuela et al., 2016). The nuclear accumulation of p50/RelA dimer is associated with early phase of inflammatory response by activating transcription of proinflammatory cytokines such as iNOS, Tnf-α, IL-1β, IL-6, and proteolytic enzymes in microglia (Kaminska et al., 2016). The production of mature p50 and p52 are processed by proteasome cleavage of NF-κB precursors (Harhaj and Dixit, 2012; Collins et al., 2016). The activation of IRF and NF-κB transcription factor pathways are important initial steps involved in immune activation.

In cultured microglial cells, signaling through the prostaglandin-E2 (PGE2) EP4 receptor modulates genes enriched in targets of IRF1, IRF7, and NF-κB transcription and attenuated levels of Aβ-induced inflammatory factors and potentiated Aβ phagocytosis (Kaminska et al., 2016). In microglia treated with LPS, IRF7 expression was observed to increase, and IRF7 knockdown was found to decrease phosphorylation of STAT1 and expression of LPS-induced genes (Zhao et al., 2017). Similarly, IRF8 deficient microglia observed to have reduced microglial marker IBA1 expression, less elaborated processes, decreased phagocytic capacity and showed less proliferative potential in mixed glial culture (Horiuchi et al., 2012). Overexpression of IRF8 is reported to promote expression of inflammatory genes whereas its deficiency prevents expression of these genes in microglia culture from the spinal cord. In peripheral nerve injury, IRF8 showed elevated expression level and implicated in regulating microglial migration (Masuda et al., 2012, 2014).

## 7. Microglia and their involvement in neuroinflammation

**Figure 2:**
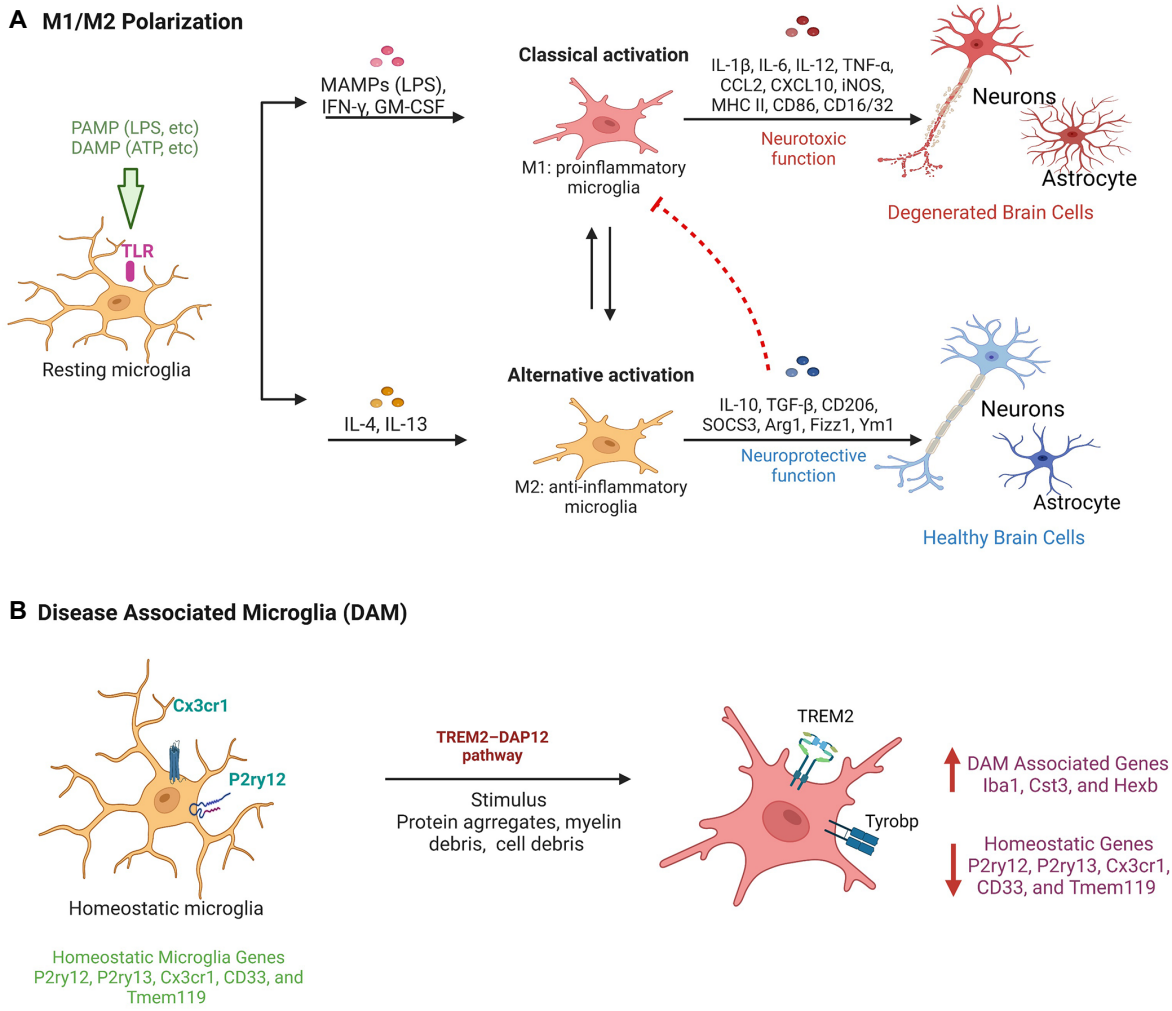
Microglial activation pathways in neuroprotection and neurodegeneration. **(A)** Classical Pathway: PAMPs/DAMPs activate TLRs such as TLR-4 on resting microglia and result in a phenotypic transition to M1 type microglia and secrete pro-inflammatory factors having neurotoxic functions. Alternative pathway: Factors such as IL-4 and IL-13 modulates the transition of microglia to M2 phenotype and provide neuro-protective function by secreting anti-inflammatory cytokines. **(B)** Diagrammatic representation showing molecular changes in homeostatic microglia leads to their conversion in disease associated microglia phenotype during neurodegeneration.

Growing evidence implicates microglia as key regulators of neuroinflammation, shedding light on their role in pathological processes. Studies highlight the various activation mechanisms of brain microglial cells. One such mechanism is classical activation, in which microglia secrete pro-inflammatory factors such as Interleukin-1 (IL-1), Interleukin-6 (IL-6), and TNF-α in response to an insult, in addition to an increase in the production of NO and ROS. These factors damage the surrounding neuronal cells (London et al., 2013). Microglia cells of M1 phenotype are the first line of defense because they can eliminate invading pathogens, such as bacteria and viruses, by recognizing pathogen-associated molecular patterns (PAMPs; Orihuela et al., 2016). PAMPs activate Toll-like receptors (TLRs) on microglia, specifically TLR4 to induce the production of proinflammatory cytokines (Fiebich et al., 2018). Impacts of TLR4 have been observed in neurodegenerative diseases such as AD and PD, and chronic neuroinflammation after stroke and spinal cord injury (Pascual et al., 2021). Microglia also adopts an alternative activation pathway known as the M2 phenotype triggered by Interleukin-4 (IL-4) or Interleukin-13 (IL-13), resulting in the secretion of anti-inflammatory cytokines such as Interleukin-10 (IL-10; Orihuela et al., 2016; Amo-Aparicio et al., 2021). The phenotypic changes of microglia cells from a pro-inflammatory to an anti-inflammatory state ensures the clearance of debris and deposition of extracellular matrix for tissue repair (Arcuri et al., 2017). Otherwise, the production of proinflammatory cytokines, NO, and ROS would be elevated. This can result in progressive cell death and damage to the tissues. However, this coordination between the two phenotypes of microglia is altered during chronic activation of microglia and thus causes inflammation in neurodegenerative diseases (Figure 2A).

Furthermore, transcriptome studies have demonstrated that activation of microglia is variable, suggesting that M1 (Ramified cells) and M2 (amoeboid cells) signify a range of activation patterns instead of just a separate cell subtypes (Correale, 2014). The M1/M2 model alone is reported to lack accuracy to explain microglia activation and its heterogeneity *in vivo* (Colonna and Butovsky, 2017). The report also suggests microglia heterogeneity in pineal gland (Muñoz, 2022). Consequently, there is no standard morphological classification, which makes it difficult to relate data from various independent studies and avoids impartial quantification of pathological status and therapeutic effects (Paolicelli et al., 2022). Further, in neurodegenerative conditions such as AD, ALS, aging a comprehensive single-cell RNA analysis of CNS immune cells identified disease-associated microglia (DAM), a subpopulation of microglia with a distinct transcriptional and functional signature. DAM is associated with the expression of neurodegenerative disease-related genes (Figure 2B). DAM are immune cells that express typical microglial markers such as Cst3, Iba1, and Hexb, concurrently with the downregulation of “homeostatic” microglial genes, such as Cx3cr1, Tmem119, P2ry12, P2ry13, and CD33 (Butovsky et al., 2014). DAM also exhibits upregulation of genes involved in lipid metabolism, phagocytic and lysosomal, pathways such as Apoe, Ctsd, Lpl, Tyrobp, and Trem2, which are known AD risk factors. DAM can be activated by a variety of stimuli, including myelin debris, protein aggregates, and cell debris, *via* the TREM2–DAP12 pathway, and can contribute to the apoptotic cells clearance, inflammatory cytokines, and myelin debris (Deczkowska et al., 2018). Therefore, the discovery of DAM in different neurodegenerative diseases paved the way for the development of a therapy targeting the universal and intrinsic mechanism for combating neuronal death that is shared by multiple neurodegenerative diseases. However, to make effective use of their therapeutic potential, it will be necessary to conduct extensive research to better comprehend the microglia phenotype, disease specific microglia characterization in CNS pathology.

### 7.1. Microglia and Alzheimer’s disease

**Figure 3:**
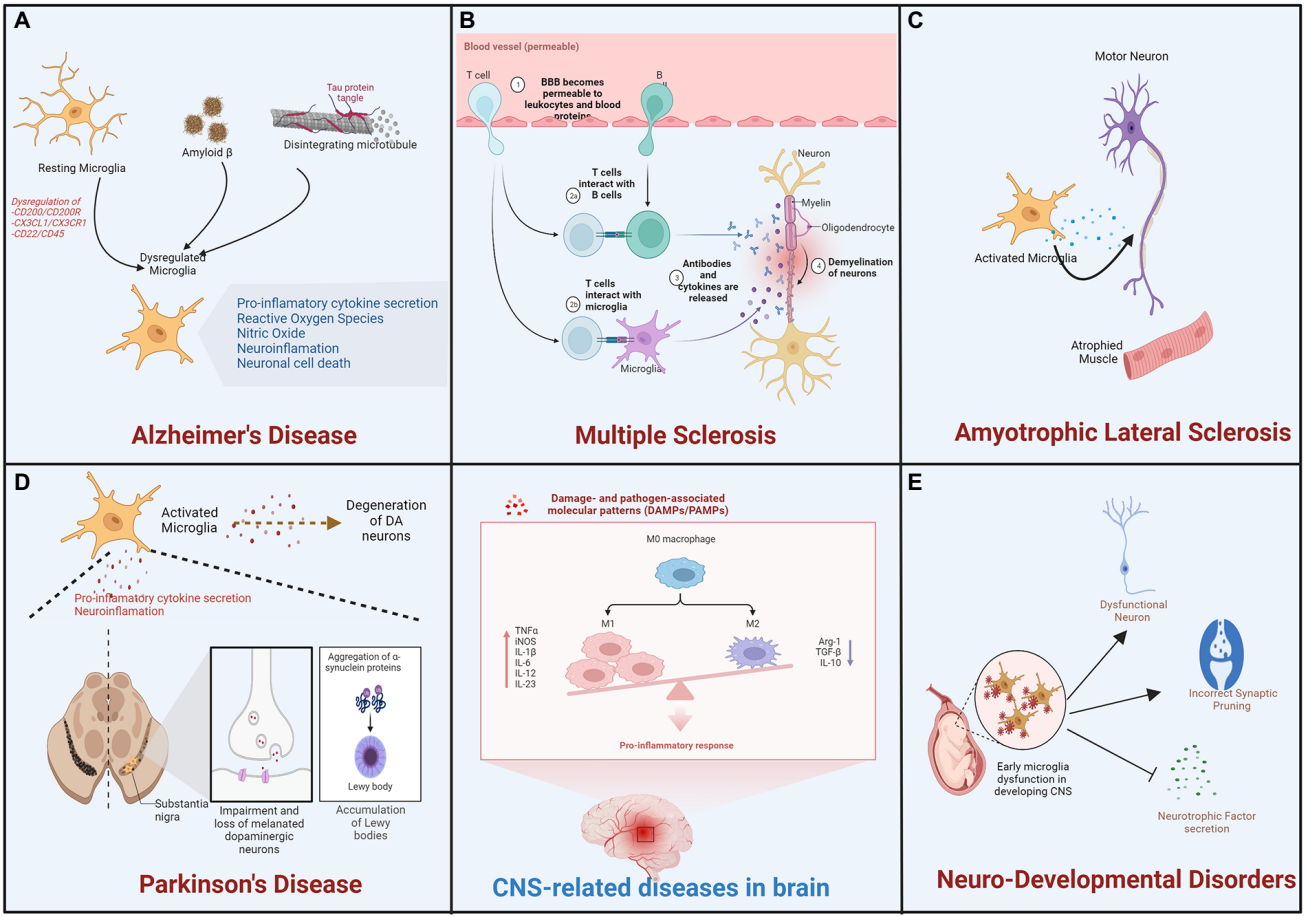
Microglial activation is involved in the progression of neurodevelopmental and degenerative diseases. During pathological processes, alternation in microglial function and activation process is involved in neurodegenerative diseases such as **(A)** Alzheimer’s disease (AD), **(B)** Multiple Sclerosis (MS), **(C)** Amyotrophic Lateral Sclerosis (ALS), and **(D)** Parkinson’s Disease (PD) as well as **(E)** neurodevelopmental diseases.

Alzheimer’s disease (AD) is the most prevalent neurological disorder, characterized by progressive cognitive decline and memory loss in most patients. It affects approximately 1 in 9 people aged 65 and over (10.7%). Alzheimer’s dementia prevalence increases with age: 5% of those aged 65 to 74, 13.1% of those aged 75 to 84, and 33.2% of those aged 85 and older have Alzheimer’s dementia (2022 Alzheimer’s disease facts and figures, 2022). Mostly during the early stages of AD, beta-amyloid (Aβ) protein secreted from neurons misfolds and forms senile plaques in the extracellular space of neuronal cells that leads to the formation of neurofibrillary tangles that is an aggregate of hyperphosphorylated Tau protein (Murphy and LeVine 3rd, 2010). An association between an immune/microglial gene network and AD neuropathology has also been reported (Zhang et al., 2013). In this condition, activation of microglia plays dual role as acute microglial activation reduces Aβ accumulation by increasing phagocytosis or clearance (Wang et al., 2015). In contrast, chronic microglial activation contributes to neurotoxicity and synaptic loss (Lull and Block, 2010), *via* the activation of multiple proinflammatory cascades (Figure 3A). Remarkably, multiple studies on AD patients and mouse models have demonstrated that activated microglia, immunoglobulins, and complement components are closely associated with Aβ deposits in the brain. It has been demonstrated *in-vitro* that Aβ1-42 activates microglia *via* CD36 and the TLR2–TLR6 heterodimer, which then expresses copious amounts of proinflammatory factors such as IL-1, TNF-α, MIP-1, and MCP-1 (Mizuno, 2012). In addition, Aβ protein stimulated conditioned media from microglia leads to increased iNOS levels and nitrotyrosine immunoreactivity (Combs et al., 2001). Further, NO synthesis inhibition using L-NMMA can ameliorate spatial memory impairment in AD (Ren et al., 2022). Furthermore, elevated levels of chemokines and proinflammatory cytokines in cerebrospinal fluid (CSF) confirm the pathological role of microglia in AD patients (Wang et al., 2015). Apart from neuroinflammation, inflammation and systemic infections have also been associated with an increased risk of developing AD. Further, microglial activation is also responsible for the hyperphosphorylation of tau and the formation of fibrillary cells through the CX3CR1 pathway. Chemokine receptor CX3CR1 is predominantly expressed on microglia and maintains the quiescent state of microglia by binding to its ligand, fractalkine (CX3CL1), which is expressed in both soluble and membrane-bound forms in neurons. Tau competes with CX3CL1 for interaction with its receptor, CX3CR1, in the AD brain, where fractalkine expression is reduced. Particularly, deletion of CX3CR1 in microglia increases IL-1 secretion and tau fibrillary tangle formation (Pawelec et al., 2020). Microglia depletion has been reported to inhibit tau proliferation, indicating that microglia actively contribute to tau pathology during AD pathogenesis (Asai et al., 2015). In addition, microglia express pattern recognition receptors (PRRs), an innate immune cell receptor that responds to molecular patterns associated with danger or pathogens (DAMPs or PAMPs), i.e., exogenous pathogenic molecules. Additionally, PRRs bind to diverse Aβ species with varying affinities. Recent research has demonstrated that, in the human AD cortex under definite conditions, Aβ species of high-molecular-weight break down into small oligomeric Aβ species which are neurotoxic in nature, and *in vivo* administration of this species to mice activates microglia. The activated microglia get identified by elevated levels of CD68 and diminished ramification. *In vitro* study suggests that Aβ by binding to PRRs, such as receptors for advanced glycation products (RAGE), toll-like receptors (TLRs), and scavenger receptors activates microglia (Wilkinson and El Khoury, 2012). Importantly, proinflammatory molecules such as TNF-α and IL-1, and other inflammatory intermediates increased production leads to compromised microglial phagocytosis upon PAMP or DAMP binding to PRRs. It appears that the expression profile of cytokines from microglia influences the phagocytotic index of microglia. Further, greater Aβ load and phagocytic index was also observed in microglia that produced IL-1 and IL-1R (Sarlus and Heneka, 2017). Interestingly, genome-wide association studies (GWAS) have reported 38 genetic loci found strongly associated with the risk of late-onset Alzheimer’s disease (LOAD). Many of them are related to neuroinflammation and are observed to be microglial cells specific such as ε4 isoform of the ApoE, Trem2, Cr1, Cd33, or Abca7, etc. (Neuner et al., 2020; Wightman et al., 2021). This provides additional support for the hypothesis that microglia reactions are likely a cause as well as a consequence of AD. To explore these functions and the association of microglial genes with disease states, however, in-depth research is necessary.

### 7.2. Microglia and multiple sclerosis

Multiple sclerosis (MS) affects approximately 2.8 million people worldwide, according to the most comprehensive global study to date. Since 2013, the Atlas of Multiple Sclerosis reveals that the number of people living with MS has increased in every region. The report indicates that 85% of people are initially diagnosed with relapsing–remitting MS, which is characterized by periods of relapse and remission, while 12% are initially diagnosed with progressive MS (Goldenberg, 2012; Klineova and Lublin, 2018). The MS is a chronic inflammatory and neurodegenerative disease of the central nervous system (CNS) that affects the white and gray matter characterized as focal lesions of inflammation, axonal loss, gliosis, and demyelination (Lassmann, 2018). It is regarded as a multifaceted, heterogeneous disease with varying patterns and mechanisms of tissue injury that are frequently difficult to treat. The course of disease for the remaining 3% is unknown at the time of initial diagnosis. In the early stages of multiple sclerosis, brain biopsies revealed subcortical and cortical demyelination and the presence of phagocytic cells (Popescu et al., 2013). As measured by the expression of the microglia-specific marker TMEM119, which is absent in macrophages, this pool of phagocytic cells in MS lesions consists of approximately 40% microglia cells (Wang et al., 2019).

Activated microglia were observed in animal models of MS, and experimental autoimmune encephalomyelitis (EAE). They were critical to the inflammation of CNS, demyelination and remyelination processes of multiple sclerosis (Robinson et al., 2014). Remyelination is the process of myelin regeneration that occurs concurrently with or after demyelination and it is characterized by the appearance of myelin around the axon (Chari, 2007). Clearance of debris and proliferation of oligodendrocytes are essential remyelination processes, followed by recruitment and proliferation of OPCs, which differentiate into mature myelinating oligodendrocytes (Bradl and Lassmann, 2010). Through the expression of anti-inflammatory molecules, phagocytosis of debris, and tissue repair, microglia promote remyelination. Microglia secrete factors such as TNF-α, IGF-1, and FGF-2 that promote oligodendrocyte proliferation (Kalafatakis and Karagogeos, 2021). IL-4 injection increased oligodendrocyte proliferation in the spinal cord of EAE mice, suggesting the potential involvement of microglia/macrophages in remyelination (Luo et al., 2017). In another similar study, oligodendrocyte differentiation in cerebellar slices was improved *in-vitro* by culture in M2 microglia-conditioned medium and decreased *in-vivo* after M2 microglia depletion; blocking of activin A secreted by M2 microglia inhibited oligodendrocyte differentiation during remyelination (Kalafatakis and Karagogeos, 2021). During the early phase of multiple sclerosis, however, remyelination is impaired because M2 microglia cells (neuroprotective) acquire a different phenotype (neurotoxic microglia) and secrete proinflammatory molecules that can damage the myelin sheath and contribute to oligodendrocyte loss (Luo et al., 2017; Kalafatakis and Karagogeos, 2021). Crosstalk between these activated neurotoxic microglia and other immune cells leads to the activation of T cells during demyelination and remyelination in multiple sclerosis (Matejuk et al., 2021; Figure 3B).

### 7.3. Microglia and amyotrophic lateral sclerosis

A degenerative disorder affecting motor neurons (MNs) of the cerebral cortex, brainstem, and spinal cord, amyotrophic lateral sclerosis is also known as Lou Gehrig’s disease in the United States. It also results in the death of upper and lower motor neurons, which control voluntary muscle contractions. This causes symptoms including muscle stiffness and twitching, limb weakness due to a gradual reduction in muscle size, and difficulty swallowing or speaking (Zarei et al., 2015). In addition, up to 50 % of patients develop frontotemporal dementia (FTD), which is characterized by progressive degeneration of the frontal and temporal lobes, behavioral and personality changes, and a decline in language and executive function (Ferrari et al., 2011). In the late stages of the disease, the weakened diaphragm and intercostal muscles typically result in respiratory failure and death. PET analysis of a small cohort of amyotrophic lateral sclerosis (ALS) patients has demonstrated the activation of microglia in ALS patients. This study revealed diffuse microglial activation in both motor and extra-motor regions of the brain (Muzio et al., 2021). In addition, studies on human postmortem tissues and mouse models of ALS have revealed an increase in activated microglia in regions of the brain with neuronal loss (Figure 3C). In addition to the altered state of microglia observed in ALS, evidence also suggests that the disease is associated with microglial degeneration (Brites and Vaz, 2014). In transgenic mutant SOD1G93A, with aggregated SOD1 also known as mice model of ALS leads to degeneration of mononuclear phagocytes including microglia and infiltrating monocytes in brain (Brites and Vaz, 2014; Liu and Wang, 2017). In postmortem ALS spinal cord tissue, upregulation of microglial receptors including toll-like receptors TLR2 and TLR4 and co-receptor CD14 has also been reported (Liu and Wang, 2017). NF-kB is a master regulator of inflammatory responses and has been associated with microglial involvement in ALS models (Frakes et al., 2014). Neurotoxic NADPH oxidase has been associated with ALS. A correlation exists between decreased NOX2 activity and increased survival in ALS patients (Marrali et al., 2014).

### 7.4. Microglia and Parkinson’s disease

Parkinson’s disease (PD) is the second most prevalent form of neurodegeneration. It manifests clinically as motor symptoms such as tremor, slow movement, rigidity, imbalance, walking etc., and a wide range of non-motor complications like dementia, mental health disorders, cognitive impairment, pain, sleep disorders, and other sensory disturbances (DeMaagd and Philip, 2015). Motor impairments, including involuntary movements (dyskinesias) and painful involuntary muscle contractions (dystonias), causing difficulty in mobility, speech, and various other aspects of life (Sveinbjornsdottir, 2016). These motor symptoms are caused by the degeneration of DA (dopaminergic) neurons located in the pars compacta of the substantia nigra (SN) in a progressive manner. The striatum is innervated by DA neurons, and their degeneration is associated with a substantial reduction in DA striatal content (Zhai et al., 2018). Loss of DA neurons is frequently associated with the formation of Lewy bodies (LB), which are aggregates of misfolded α-synuclein in the SN, but also in other brain regions (Power et al., 2017). Sustained α-synuclein burden leads to aberrant microglial activation, and targeting microglial activation states by suppressing their deleterious pro-inflammatory neurotoxicity has been proposed as a therapeutic approach for PD treatment. Similarly, increased levels of pro-inflammatory cytokines IL-1β, TNF-α and iNOS mRNA, degeneration of nigral dopaminergic neurons has been reported in Parkin deficient mice (Frank-Cannon et al., 2008; Tran et al., 2011). Aberrant microglial activation and increased astrogliosis has also been observed in aged parkin-null mice (Rodriguez-Navarro et al., 2007). Thus, loss of Parkin is also reported to influence activated microglia survival and cause chronic neuroinflammation (Dionísio et al., 2019).

Remarkably, in the postmortem PD patients SN, neuronal cell death was observed along with significant microglial activation in the affected brain region. These activated microglia reported to produce a variety of inflammatory mediators, such as NOS2, IL-6, COX2, TNF-α, and ROS, result in the long-term and continuous loss of DA neurons (Marogianni et al., 2020). In the SN of living PD patients, positron emission tomography (PET) analysis consistently detects activated microglia and significant death of dopamine (DA) neurons. In addition, reactive microglia are observed in brain regions other than SN, indicating that microglia play a global role in the development of PD (Belloli et al., 2020). PET imaging has confirmed potent activation of microglia in a 6-hydroxydopamine (6-OHDA)-induced PD model, and depletion of TNF-α by pharmacological neutralization or by a dominant-negative mutant should reduce the loss of dopaminergic neurons and improve behavioral performance (More and Choi, 2016). In another PD model established with 1-methyl-4-phenyl-1,2,3,6 tetrahydropyridine (MPTP), microglial activation in the SN has also been confirmed by a dramatic morphological change (Meredith and Rademacher, 2011). Significantly, deletion of TLR-4 in microglia inhibits their activation, thereby promoting the survival of DA neurons (Cui et al., 2020). Further, it was hypothesized that neurotoxic reactive species produced by microglia, such as superoxide and nitric oxide, induce cellular stress and contribute to neuronal loss in PD (Drechsel and Patel, 2008). In addition, the cerebrospinal fluid of PD patients was found to be toxic to DA neurons *in vitro* due to the high concentration of cytokines and autoantibodies against quinone proteins altered by DA oxidation (Whitton, 2007). Reports also suggest neuroprotective role of microglia in PD by initiating neuron–glia crosstalk, regulating microRNA, releasing anti-inflammatory cytokines such as IL-4, Arg1, Tgf-beta, activating multiple receptors and suppressing the expression of neurotoxic genes by modifying histone tails (Le et al., 2016). However, continuous stimulus from endogenous factors such as α-synuclein aggregates contribute to elevated inflammation in the brain and succeed the protective effects of microglia and thus drive the chronic and progressive neurodegeneration observed in PD (Le et al., 2016; Bido et al., 2021). These findings strongly suggest that inflammation mediated by microglia plays a pathogenic role in PD (Figure 3D).

### 7.5. Microglia and neurodevelopmental disease

Microglia are believed to play a crucial role in neurogenesis, the transformation of precursor cells into neurons. During normal neurodevelopment, microglia induce apoptosis and phagocytosis in a number of newborn neurons; however, in the adult brain, this process is restricted to neurogenic niches. In addition, *in-vivo* analysis of prenatal brain studies reveals that microglia phagocytose neural progenitor cells, which may contribute to neurodevelopmental disorders such as ID (intellectual disability), ADHD (attention-deficit/hyperactivity disorder), ASD (autism spectrum disorder), CD (communication disorders), SLD (specific learning disorder), and MDs (motor disorders), which affect 2–4 percent of the world’s population (Figure 3E). Due to the difficulty in studying embryonic systems, most studies examining the role of microglia in neurogenesis employ *in vitro* or adult neurogenesis methodologies. Vargas et al., 2005 were the first to demonstrate an inflammatory phenotype in the postmortem brains of individuals with ASD. Neuropathological examination of the cerebral and cerebellar cortices of individuals with autism spectrum disorder revealed increased microglial activation, characterized by elevated expression of MHC class II (Needleman and McAllister, 2012). In addition, increased expression of pro-and anti-inflammatory factors, including CCL2, IL-6, and TGF-β, was observed in the brain and cerebrospinal fluid (CSF). In the post-mortem brain of ASD, expression level analysis of pro-inflammatory cytokines in tissue and CSF suggest altered immune response and their link with region specific brain inflammation (Goines and Ashwood, 2013). Similarly, GWAS analysis of ASD brains showed genes related to activated microglia, immune and inflammatory functions suggesting astrocyte and microglial immune dysregulation (Davoli-Ferreira et al., 2021). Along with it, alteration in microglia morphology, spatial distribution and density was also reported in ASD brain. In addition to having a higher density in the cerebral and cerebellar cortices of ASD patients, microglia exhibit enlarged cell bodies, processes thickening and retraction. ASD-associated microglia also extend filopodia from their processes (Morgan et al., 2010; Davoli-Ferreira et al., 2021). Similar studies have demonstrated the role of microglial cells in inducing the loss of cortical gray matter in schizophrenia patients by pruning synapses, phagocytosing stressed neurons, and inhibiting the release of neurotrophic factors such as BDNF (Mallya and Deutch, 2018). In a systematic review by Trépanier et al., 2016 multiple studies reported an increase in the expression of microglial markers in the postmortem brains of schizophrenia patients as compared to control subjects. In addition, a meta-analysis conducted by van Kesteren et al., 2017 revealed an increase in the number of microglia in specific brain regions, such as the temporal cortex, in the postmortem brains of schizophrenia patients compared to those of control subjects. Nevertheless, there have been numerous contradictory studies regarding microglial cell activation and neurodevelopmental disease (NDD). To better comprehend the role of microglial cells and associated factors, patients must be subjected to a comprehensive analysis and screening.

## 8. Possible transcriptional target in microglia for regulating brain homeostasis in neurodegenerative diseases

For an extended period of time, the difficulty of microglia-specific conditional gene targeting has hindered the thorough understanding of microglia residing in the CNS and their functions. However, recent developments have produced a variety of strategies to combat non-specificity. Multiple signature genes have been proposed as being essential for microglia function. It has been discovered that the transcriptional regulator Sall1 is highly expressed in adult microglia. Sall1 controls a unique transcriptional signature permitting microglia function and fate in the regulation of homeostasis in the adult CNS. Deletion of Sall1 in microglia leads to dysregulated neurogenesis and microglia activation which further affects cognitive and behavioral function and impairs memory formation. It has been proposed that Sall1 could act as the master transcriptional regulator of housekeeping functions and the non-reactive state of microglia, which are essential for CNS homeostasis. In addition, Sall1CreER mice were reported to target microglia without targeting other MPS members (Buttgereit et al., 2016). Microglia’s development, proliferation, migration, differentiation, and survival are governed by the CSF1R, a key signaling node. CSF1R signaling was required for adult microglial survival. In the 5Xfad mouse model of AD, the CSF1R inhibitors (PLX3397 and PLX5622) deplete microglia and prevent plaque formation in brain parenchyma (Spangenberg et al., 2019; Green et al., 2020). Another CSF1R inhibitor, JNJ-40346527 (JNJ-527), inhibits microglial proliferation and reduces tau-induced neurodegeneration (Mancuso et al., 2019). Signaling of TGF-R has been linked to the formation and maintenance of microglia. Further, microglia-specific deletion of TGF-R led to a discovery that TGF-R signaling is involved in maintaining the homeostatic phenotype of adult microglia but not their survival (Zöller et al., 2018).

Modulating mitochondrial function and reactive oxygen species (ROS) levels, PGC-1α has also been implicated in the pathogenesis of numerous neurological disorders (Panes et al., 2022). Reduced PGC-1 expression was preceded by decreased expression of mitochondrial antioxidants and uncoupling proteins (UCPs) in multiple sclerosis patients, resulting in neuronal loss and neurodegeneration (Witte et al., 2013). Furthermore, PGC-1 α reported to upregulate mitochondrial antioxidants SOD2 and GPx1, reduces ROS generation, and attenuated MPTP-induced neurodegenerative processes in a PD model (Piccinin et al., 2021). PGC-1α significantly suppresses oxidative damage and the production of proinflammatory mediators in primary human astrocytes (Nijland et al., 2014). In the microglia of an animal model of acute ischemic stroke (AIS) and stroke patients, the altered expression of PGC-1α was reported. Overexpression of microglial PGC-1α has been suggested to inhibit neuroinflammatory responses. PGC-1α may also stimulate mitophagy and autophagy *via* unc-51-like kinase 1 (ULK1), which inhibits hyperactivation of the NLRP3 inflammasome, thereby reducing neuroinflammation and neurological deficits (Han et al., 2021). The neuroprotective effects of PGC-1α were eliminated when autophagy and mitophagy were inhibited pharmacologically and genetically. Consequently, targeting microglial PGC-1α may be advantageous for the treatment of AIS. Microglial activation suppression is one of the well-established anti-inflammatory effects of fasting and the ketogenic diet. The activation of GPR109A by beta-hydroxybutyrate inhibits NF-kB signaling and the production of pro-inflammatory cytokines in microglia and promotes neuroprotective phenotype in microglia (Fu et al., 2015). Magnolol (MA) reduced chronic unpredictable mild stress (CUMS)-induced depressive-like behavior by inhibiting M1 microglia polarization and promoting M2 microglia polarization through Nrf2/HO-1/NLRPP signaling. Transfection of Nrf2 siRNA confirmed the role of Nrf2 in the modulation of microglia polarization by MA. In addition, the enhanced effect of MA on Nrf2 was a result of the inhibition of Nrf2 ubiquitination (Li et al., 2018; Tao et al., 2021).

Hypoxia causes oxidative stress and induction of both phosphorylation and S-glutathionylation of transcription factor STAT1 which leads to its aberrant activation in M1 microglia. Report also showed reduced hypoxia-M1 microglia phenotype upon STAT1 silencing suggesting the strong link between hypoxia-STAT1 and STAT1-microglia activation (Butturini et al., 2019). Another transcription factor STAT3, also known to regulate inflammatory gene expression and microglial activity in response to various CNS insults. Aberrant activation of STAT3 after cerebral ischemia leads to neuroinflammatory processes and promotes transcription and expression of genes involved in proinflammatory mediators including inflammatory enzymes. Following ischemic injury, STAT3 overactivation in microglia causes microglia activation and inflammatory responses in the dentate gyrus (DG) and brain cortex region (Chen et al., 2017). Similarly, STAT6 deficiency has been shown to exacerbate local inflammation, enlarges brain tissue loss, clearance of dead/dying neurons and worsens long-term functional deficits. Early after stroke, STAT6 is reported to mediate effective efferocytosis and anti-inflammatory responses of microglia and STAT6/Arg1 signaling is proposed to be a viable therapeutic target to regulate microglia functions and promote long-term favorable outcomes during brain injury (Cai et al., 2019).

TFEB (Transcription factor EB) functions as the master regulator of lysosomal function. The deacetylation of TFEB intensifies its contribution to microglial degradation of fAβ and reduces amyloid plaques in brain slices from APP/PS1 mice (Bao et al., 2016). Pyruvate kinase M2 (PKM2), an essential glycolytic rate-limiting enzyme, interacts directly with the pro-inflammatory transcription factor ATF2 (activating transcription factor 2) to link pyroptosis and glycolysis in microglia, which may represent crucial crosstalk between neuroinflammation and metabolic reprogramming and in the CNS. Treatment of brain and muscle ARNT-like 1 (Bmal1) deficient mice treated with MPTP reported significant reductions in numbers of dopaminergic neurons (DANs) in the substantia nigra pars compacta (SNpc), tyrosine hydroxylase protein level in the striatum, dopamine (DA), and 3,4-dihydroxyphenylacetic acid content, respectively indicating that Bmal1 may play a crucial role in the survival of DANs and in maintaining the normal function signaling pathway in the brain by regulating microglia-mediated neuroinflammation. Disruption of the basic helix–loop–helix (bHLH) transcription factor lymphoblastoid leukemia-derived sequence 1 (Lyl-1) basic helix–loop–helix domain induces an increase in the emergence of primitive macrophage progenitors followed by a defect in their differentiation. These defects are linked to a disruption in the expression of gene sets associated with neurodevelopment. The microglia number was also found to decrease in the developing brain under Lyl-1 deficiency (Wang et al., 2021).

MEF2a (Myocyte enhancer factor 2a), that is exclusively expressed in adult microglia, is essential for maintaining the microglial phenotype and establishing the epigenetic landscape. MEF2a motif analysis revealed an increased proportion of adult microglia gene promoters associated with regulatory regions (Yeh and Ikezu, 2019). Similarly, MEF2c (Myocyte enhancer factor 2c) is diminished in aged mice due to increased expression of type-1 interferon (IFN-1) in 5x FAD mouse model representing early microglial changes in AD related pathology (Xue et al., 2021). TNF-α, a proinflammatory cytokine associated with aging, induces IBA1 intensity in immunohistochemistry study in MEF2c-deficient mice (Deczkowska et al., 2017). Following LPS stimulation *in vitro*, microglia lacking MEF2c secreted more proinflammatory cytokines and displayed less social preference (Deczkowska et al., 2017). Hematopoietic precursors express a high level of Runt-related transcription factor 1 (RUNX1). The enrichment of the RUNX1 binding motif in the enhancer landscapes of adult microglia suggests a possible role in the maintenance of the adult microglial phenotype (Lopez-Atalaya et al., 2018). V-maf musculoaponeurotic fibrosarcoma oncogene homolog B (MAFB) reported to be specifically expressed in adult microglia. During aging, MAFB expression is observed to increase significantly. Further, it is known to play a key role in the maturation and differentiation of adult microglia, and is proposed to regulate microglia response under stressful or pathological conditions. MAFB regulates expression of immune and viral genes and promotes anti-inflammatory phenotypes. In the presence of GM-CSF, MAFB-deficient microglia exhibit increased self-renewal and decreased P2ry12 and Ccl2 expression (Koshida et al., 2015). Hhex is reported to negatively regulate microglia inflammation-related genes, and TLR2/4 activation decreases Hhex, thereby promoting TLR4-mediated neuroinflammation (Sakate et al., 2022).

## 9. Epigenetic factors and their key role in the regulation of microglia function in normal and disease conditions

In microglia, lineage-determining transcription factors including PU.1 and SALL1 form complexes with gene enhancers and become co-activators to regulate the transcription of targeted genes. The tightly regulated acetylation of histone H4 on the promoter and intron-1 region of the PU.1 locus was reported essential for allowing the interaction between RNA polymerase II and locus (Laribee and Klemsz, 2005). Further, it was observed that histone deacetylase (HDAC) activity is essential for association of RNA polymerase II with PU.1 promoter (Laribee and Klemsz, 2005). Further, altering catalytic activity of HDACs by treatment of valproic acid and vorinostat are shown to regulate microglial transcriptomes *via* PU.1 suppression (Dragunow et al., 2006; Rustenhoven et al., 2018). Another epigenetic regulator, Runx1t1 (Runt-related transcription factor 1; translocated to, 1) also interacts with HDACs, epigenetically regulates Cdk4 and LAT controls the proliferation and nitric oxide production of microglia (Baby et al., 2014). Inhibiting HDAC1 and HDAC2 has been shown to suppress inflammatory response of chronically activated microglia, thus proposed as a therapeutic approach toward management of neuroinflammation (Durham et al., 2017). In mice, ablation of Hdac1 and Hdac2 leads to impaired microglia development during the prenatal stage whereas they did not have any effect on microglia survival during homeostasis in the adult stage. Deletion of Hdac1 and Hdac2 from microglia of AD mice model lead to enhanced phagocytosis, decreased amyloid deposition and improved cognitive impairment (Datta et al., 2018). Further, the presence of active histone H3 at the lysine 4 (H3K4) region, in both wild-type and MGnD microglia indicates that disease-associated regions primed in MGnD can also be primed in homeostatic microglia. Histone methylation occurring on the amino (N) terminal tail of the core histone H3 (H3K27me3) is known to be associated with the downregulation of nearby genes *via* the formation of heterochromatin regions and thus regulates the efficient clearance of apoptotic cells and debris by microglial cells (Petralla et al., 2021). It was observed that epigenetic modification of H3K27me3 leads to suppression of clearance-related genes and this affects the region-dependent phagocytic activity of microglia (Ayata et al., 2018).

In *Mecp2* null mice, microglia activation and loss have been observed with disease progression (Cronk et al., 2015). In Rett Syndrome, Mecp2 deficiency is reported to cause microglial activation and dysfunction (Kahanovitch et al., 2019). Mecp2 regulates glutamate synthesis and mitochondria function in microglia by acting as a transcriptional repressor of the major glutamate transporter, SNAT1 (Jin et al., 2015). Mecp2 depletion causes an increase in histone acetylation at enhancer regions of Fkbp5, a canonical glucocorticoid target gene, and recruitment of nuclear receptor co-repressor 2 (NCOR2) and HDAC3 complex leading to dysregulation of genes involved in glucocorticoid signaling, hypoxia response and inflammatory responses, suggesting Mecp2 is critical for maintaining immune cell function including microglia (Yeh and Ikezu, 2019).

Reports also indicate factors that can lead to epigenetic modification in microglia are peripheral immune stimulation and cerebral beta-amyloidosis. The activation of HIF-1a and mTOR pathways in response to cerebral beta amyloidosis cause transcriptional and functional alterations linked with increased immune response genes and inflammation in MGnD. In LPS-treated amyloid-β precursor protein (APP) transgenic mice, H3K4me1 increased levels in putative enhancers reported related to phagocytic function (Wendeln et al., 2018). Therefore, more precise epigenetic studies are necessary for the genetic targeting of specific HDACs in microglia. Apart from histone modifications, microRNAs (miRNAs) are also involved in regulating the CNS epigenetic landscape. miRNAs are small non-coding RNA molecules, known to regulate gene expression. In brain, mRNA and miRNA microarray assays analysis identified miR-155 and miR-124 as regulator of development of microglial cells, microglia activation pathways and microglia quiescence in the CNS (Ponomarev et al., 2011; Cardoso et al., 2012).

## 10. Conclusion and future prospective

Microglia are brain specific immune cells, interacts with neurons, astrocytes, oligodendrocytes and play an important role in maintenance of brain homeostasis *via* performing regular immunological surveillance, regulating neuroinflammation, maintaining synaptic plasticity, cognition etc. To better understand the role of microglia in brain homeostasis, further research is required to decipher the gene-specific functions of microglia and their interconnections with other brain cells. Further, microglia physiology is also critical for progression of neurodegenerative diseases. Despite the many advancements made in recent years, there are still many unanswered questions. Exciting new pharmacological agents that target not only the deleterious functions of microglia, but also mechanisms that promote endogenous repair, are likely to become available in the near future. Overall, future studies will facilitate clinical advancement and investigation of the role of microglia in a variety of nervous system functions.

## Author contributions

SM designed the study. SM and SG did a literature survey, wrote the part of the manuscript, and compiled the manuscript. RM reviewed and edited the manuscript. All authors contributed to the article and approved the submitted version.

## Funding

SM acknowledges financial support from the Institute of Eminence, University of Delhi (IOE/2021/12/FRP) and University of Delhi, Delhi-110007.

## Conflict of interest

SG was employed by Tech Cell Innovations Private Limited.

The remaining authors declare that the research was conducted in the absence of any commercial or financial relationships that could be construed as a potential conflict of interest.

## Publisher’s note

All claims expressed in this article are solely those of the authors and do not necessarily represent those of their affiliated organizations, or those of the publisher, the editors and the reviewers. Any product that may be evaluated in this article, or claim that may be made by its manufacturer, is not guaranteed or endorsed by the publisher.
